# Supplementary material for: Production of Active Recombinant Hyaluronidase Inclusion Bodies from Apis mellifera in E. coli Bl21(DE3) and characterization by FT-IR Spectroscopy
Source: Int J Mol Sci. 2020 May 29;21(11):3881. doi: 10.3390/ijms21113881 (PMC7313074; doi:10.3390/ijms21113881)
Supplement: Supplementary file 1 [file ijms-21-03881-s001.pdf]

Production of active recombinant Hyaluronidase Inclusion Bodies from Apis mellifera in E. coli Bl21(DE3) and characterization by FT-IR Spectroscopy

Andreas Schwaighofer[3]; Sarah Ablasser[1]; Laurin Lux[3], Julian Kopp[1]; Christoph Herwig[1]; Oliver Spadiut[2]; Bernhard Lendl[3] and Christoph Slouka[2,*]

Supplementary Infiormation

Transformation of BL21(DE3)

Sequence:

CAT

ATGAGCCGTCCGCTGGTTATTACCGAAGGCATGATGATTGGCGTTCTGCTGATGCTGGCACCGATTAATGCCCTGCTGC

TGGGTTTTGTTCAGAGCACCCCGGATAATAATAAGACCGTGCGTGAATTCAATGTGTATTGGAATGTGCCGACCTTTAT

GTGTCATAAATATGGCCTGCGTTTTGAAGAAGTTAGCGAAAAATATGGTATCCTGCAGAATTGGATGGATAAATTTCGC

GGTGAAGAAATTGCAATTCTGTATGATCCGGGCATGTTTCCGGCACTGCTGAAAGATCCGAATGGTAATGTTGTTGCCC

GTAATGGTGGCGTGCCGCAGCTGGGTAATCTGACCAAACATCTGCAGGTGTTTCGCGATCATCTGATTAATCAGATTCC

GGATAAAAGCTTTCCGGGTGTTGGTGTGATTGATTTTGAAAGCTGGCGCCCGATTTTTCGCCAGAATTGGGCCAGTCTG

CAGCCGTATAAAAAACTGAGTGTGGAAGTTGTTCGTCGCGAACATCCGTTTTGGGATGATCAGCGTGTGGAACAGGAA

GCCAAACGCCGTTTTGAAAAATATGGCCAGCTGTTTATGGAAGAAACCCTGAAAGCCGCAAAACGCATGCGTCCGGCC

GCCAATTGGGGCTATTATGCCTATCCGTATTGCTATAATCTGACCCCGAATCAGCCGAGCGCCCAGTGTGAAGCAACCA

CCATGCAGGAAAATGATAAAATGAGTTGGCTGTTTGAAAGCGAAGATGTTCTGCTGCCGAGCGTGTATCTGCGCTGGA

ATCTGACCAGTGGTGAACGTGTTGGCCTGGTTGGTGGTCGTGTTAAAGAAGCACTGCGCATTGCACGTCAGATGACCAC

CAGCCGCAAAAAAGTTCTGCCGTATTATTGGTATAAGTATCAGGATCGTCGCGATACCGATCTGAGCCGTGCAGATCTG

GAAGCAACCCTGCGCAAAATTACCGATCTGGGTGCAGATGGCTTTATTATTTGGGGTAGCAGTGATGATATTAATACCA

AAGCCAAATGCCTGCAGTTTCGTGAATATCTGAATAATGAACTGGGCCCGGCCGTGAAACGCATTGCACTGAATAATA

ATGCCAATGATCGTCTGACCGTGGATGTGAGCGTTGATCAGGTT

CTCGAG

E. coli BL21(DE3) was grown on LB agar plates [44]. For propagation, one colony from the plates was transferred into 1 mL LB broth, incubated at 37 °C overnight. 600 µL from this bacteria solution were further transferred into 44 mL LB broth in an Erlenmeyer flask and cultivated for two hours at 37 °C. For transfection, the plasmid (5 µg lyophilized) was dissolved in 50 µL ultrapure water (MQ) and diluted to a

concentration of approx. 50 ng/µL. One mL of the bacteria solution was centrifuged and washed two times with ultrapure water. 20 µL of the competent cells were mixed with 1 µL plasmid in a cuvette and transformed via electroporation at 1342 V for 6.4 ms. The cells were transferred into 1 mL LB broth and incubated for two hours at 37 °C. To check for the transformation, the cells were grown on LB agar plates containing 15 µg/µL kanamycin, using 50, 100 and 150 µL bacterial suspension, respectively. For the negative control, 100 µL bacterial suspension without plasmid were used. All plates were incubated at 32 °C overnight. Colonies grew on all plates except for the control plate. For further cultures 30 µg/µL kanamycin was used.
Single cultures were picked and eight liquid cultures were prepared using 2 mL LB broth. Four cultures were incubated at 37 °C and 30 °C, respectively. After 44 hours, three cultures incubated at 30 °C and two incubated at 37 °C showed growth. These were chosen to purify the plasmid DNA using GeneJET Plasmid Miniprep Kit from Thermo Scientific, resulting in 30 µL plasmid DNA. The plasmid concentrations were determined via NanoDrop spectrophotometer (Thermo Scientific, Waltham, MA, USA). Another 5 liquid cultures were prepared using 5 mL LB broth with 5 µL of the respective bacteria solutions. After incubation, the plasmid DNA was purified again (using MQ instead of elution buffer) and the concentrations were determined.) The bacteria culture having the highest plasmid DNA concentration (211.9 ng/µL) was chosen to be sequenced (Microsynth, Switzerland). Finally, Cryo stocks were prepared by mixing 1 mL bacteria solution with 175 µL 75% glycerol each and stored at -80 °C as starting material for fermentations.

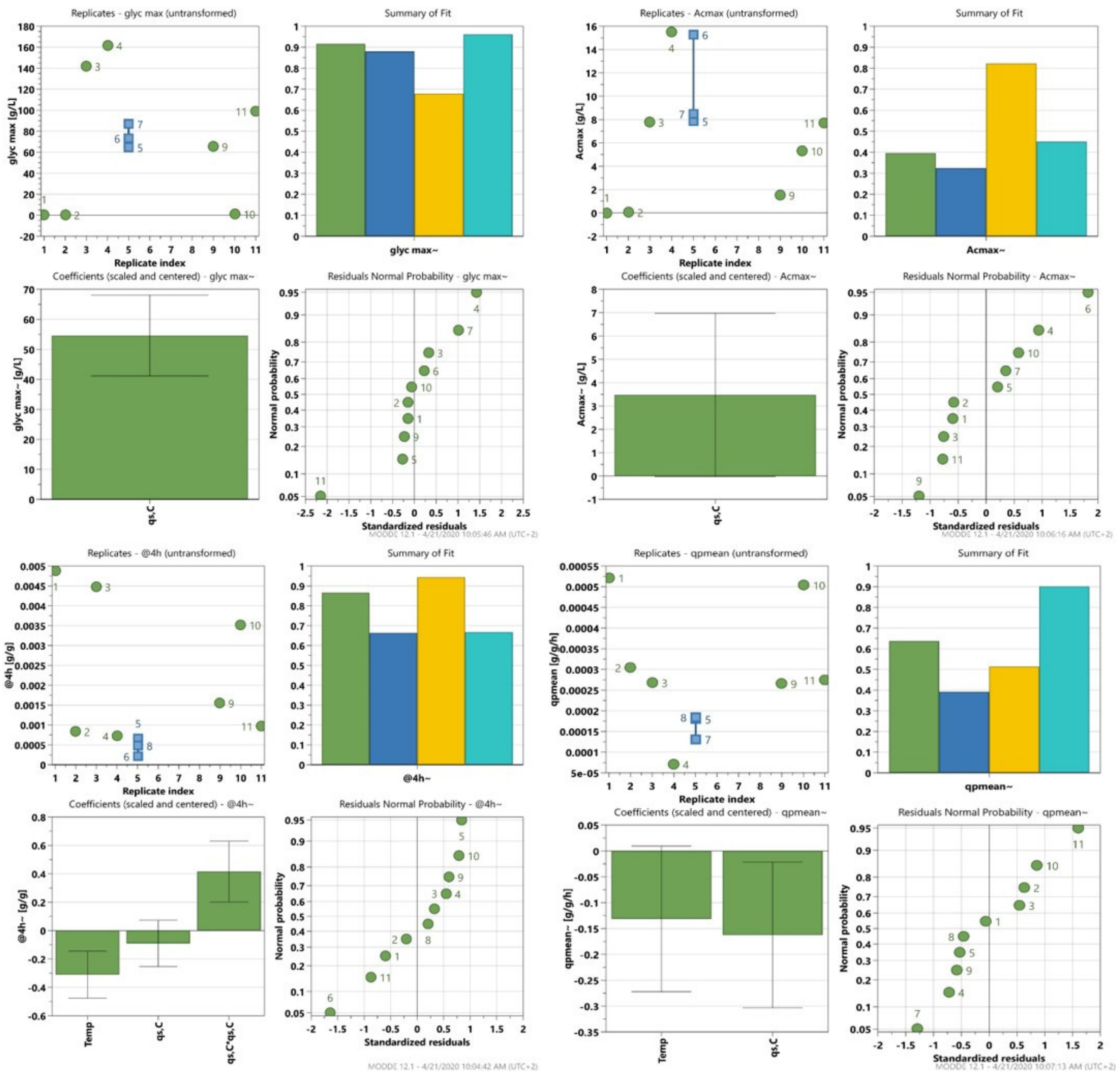

S1: Model evaluation by Modde 12 for contour plots given in Figure 3.

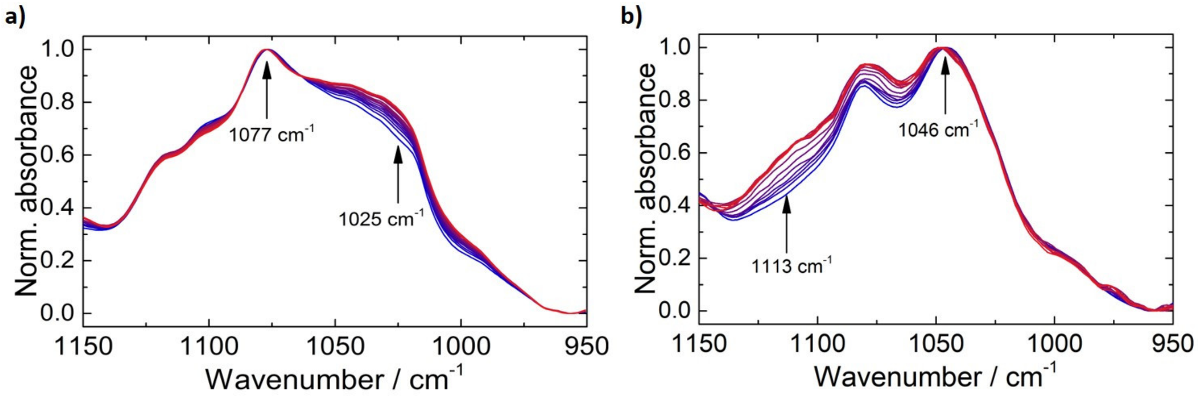

S2: a) FT-IR spectra of the enzymatic reaction at an IB concentration of 12.6 mg/mL. The blue spectrum indicates the begin of the reaction and the red spectrum was taken 40 minutes after its start, **b)** analyzed IR bands for degradation of hyaluronan. Different bands showed high changes compared to IBs;
